# Atorvastatin Improves Hepatic Lipid Metabolism and Protects Renal Damage in Adenine-Induced Chronic Kidney Disease in Sprague-Dawley Rats

**DOI:** 10.1155/2019/8714363

**Published:** 2019-11-05

**Authors:** Hardik Ghelani, Valentina Razmovski-Naumovski, Vamsi Inampudi, Dennis Chang, Srinivas Nammi

**Affiliations:** ^1^School of Science and Health, Western Sydney University, Sydney, NSW 2751, Australia; ^2^NICM Health Research Institute, Western Sydney University, Sydney, NSW 2751, Australia; ^3^South Western Sydney Clinical School, School of Medicine, University of New South Wales, Kensington, NSW 2052, Australia

## Abstract

**Objective:**

Chronic kidney disease (CKD), including nephrotic syndrome, is a major cause of cardiovascular morbidity and mortality. The literature indicates that CKD is associated with profound lipid disorders largely due to the dysregulation of lipoprotein metabolism which further aggravates the progression of kidney disease. The present study sought to determine the efficacy of atorvastatin treatment on hepatic lipid metabolism and renal tissue damage in CKD rats.

**Methods:**

Serum, hepatic and faecal lipid contents and the expression and enzyme activity of molecules involved in cholesterol and triglyceride metabolism, along with kidney function, were determined in untreated adenine-induced CKD, atorvastatin-treated CKD (10 mg/kg/day oral for 24 days) and control rats.

**Key Findings:**

CKD resulted in metabolic dyslipidaemia, renal insufficiency, hepatic lipid accumulation, upregulation of 3-hydroxy-3-methyl-glutaryl-coenzyme A (HMG-CoA) reductase, acyl-CoA cholesterol acyltransferase-2 (ACAT2) and the downregulation of LDL receptor protein, VLDL receptor, hepatic lipase, lipoprotein lipase (LPL), lecithin–cholesterol acyltransferase (LCAT) and scavenger receptor class B type 1 (SR-B1). CKD also resulted in increased enzymatic activity of HMG-CoA reductase and ACAT2 together with decreased enzyme activity of lipase and LCAT. Atorvastatin therapy attenuated dyslipidaemia, renal insufficiency, reduced hepatic lipids, HMG-CoA reductase and ACAT2 protein abundance and raised LDL receptor and lipase protein expression. Atorvastatin therapy decreased the enzymatic activity of HMG-CoA reductase and increased enzymatic activity of lipase and LCAT.

**Conclusions:**

Atorvastatin improved hepatic tissue lipid metabolism and renal function in adenine-induced CKD rats.

## 1. Introduction

Chronic kidney disease (CKD) encompasses a spectrum of different pathophysiological processes associated with abnormal kidney function and a progressive decline in glomerular filtration rate. CKD is a serious health problem, and its prevalence is increasing worldwide due to a rise in the prevalence of systemic diseases that damage the kidney [1, 2]. CKD results in the profound alteration of lipid metabolism which is manifested by hypercholesterolaemia, hypertriglyceridemia, reduced high density lipoprotein (HDL) cholesterol, impaired HDL maturation and decreased HDL antioxidant and anti-inflammatory properties [3–5]. Furthermore, the associated dyslipidaemia has been shown to contribute to the progression of kidney disease [6, 7] and therefore, the treatment of dyslipidaemia in patients with CKD should ideally confer benefits in terms of both reducing cardiovascular risk and retarding the progression of renal disease [8].

Cellular cholesterol homeostasis is regulated by the influx, biosynthesis, catabolism and efflux of cholesterol. An alteration in these processes can result in the conversion of macrophages, mesangial cells and vascular smooth muscle cells into foam cells [9]. Cholesterol synthesis in the liver is mediated by several independent pathways including hydroxy-3-methyl-glutaryl-coenzyme A (HMG-CoA) reductase, a rate-limiting enzyme in cholesterol biosynthesis, whereas cholesterol catabolism is primarily mediated by the LDL receptor [10]. Acyl-CoA cholesterol acyltransferase (ACAT) 2, a liver specific acyltransferase, catalyses the esterification of cholesterol for intracellular storage in the liver. Furthermore, ACAT is responsible for packaging and releasing cholesterol ester in VLDL and chylomicrons in the intestine. In the vascular and renal tissues, ACAT plays a central role in foam cell formation which represents the earliest lesion in atherosclerosis and glomerulosclerosis [11, 12]. Lipase is an important enzyme expressed in a variety of tissues, including liver, skeletal muscle, heart and adipose tissue and catalyses the hydrolysis of triglycerides contained in the triglyceride-rich lipoproteins, such as VLDL and chylomicrons [13]. Thus, lipase deficiency has been shown to markedly elevate serum triglycerides and VLDL levels and impair chylomicron clearance in CKD patients [14] and in experimentally-induced CKD animals [15]. CKD is consistently associated with reduced plasma HDL cholesterol concentration, impaired maturation of cholesterol ester-poor HDL-3 (nascent HDL) to cholesterol ester-rich HDL-2 (mature HDL), increased HDL triglycerides and depressed plasma apoA-I [16]. These abnormalities are primarily due to CKD-induced dysregulation of several important proteins such as lecithin-cholesterol acyltransferase (LCAT) [17, 18], scavenger receptor class B type 1 (SR-B1) [19–21] and ATP binding cassette A1 (ABCA1) [22].

Statins, or HMG-CoA reductase inhibitors, are the most common, clinically used lipid-lowering drugs and have been demonstrated to be effective in reducing LDL levels and cardiovascular mortality in the general hyperlipidaemic population. Considering the clinical benefits of statins in general nonuremic population, a similar benefit also for patients with CKD may be assumed. Furthermore, atorvastatin showed a renoprotective effects in diabetic mice via the downregulation of RhoA and upregulation of Akt/GSK3 signaling pathway in kidney [23]. Shibashaki et al. [24] showed that pitavastatin reduces inflammation within atherosclerotic lesions in mice with late-stage renal disease. Furthermore, atorvastatin attenuates kidney function impairment, proteinuria and mesangial cell proliferation in bovine gamma-globulin rat model of chronic glomerulonephritis which is the major cause of end-stage kidney disease [25]. Although there are several observational studies to evaluate the effects of statins on CKD-induced dyslipidaemia and associated cardiovascular death in humans, there are few preclinical studies of statins on CKD-induced dyslipidaemia [26]. Furthermore, most of the available literature data on the effects of statins in CKD patients are extrapolated from post hoc analyses of large prospective interventional trials carried out on subjects with established cardiovascular diseases, but not specifically addressing CKD patients [27–29]. Several large clinical trials showed a conflicting result on renoprotective effects of statin in CKD populations [30]. Nevertheless, the chronic administration of statin markedly improved circulatory lipids abnormality [17, 31] and also can ameliorate markers of endothelium dysfunction and offers a significant protective effect against the development of renal failure in 5/6 nephrectomised CKD rats [32]. Thus, the present study explored the effects of atorvastatin on the lipid metabolism and renal function in adenine-induced CKD rats.

## 2. Materials and Methods

### 2.1. Chemicals Used

Atorvastatin was purchased from Sigma (St. Louis, MO, USA). Cholesterol, triglyceride, HDL cholesterol, albumin, creatinine and urea nitrogen diagnostic kits were obtained from PM Separations (Capalaba, DC, USA), while the diagnostic kits for nonesterified free fatty acids (NEFA) and bile acids were obtained from Wako Diagnostics (Osaka, Japan). Electrophoresis and electroblotting consumables were purchased from Bio-Rad (Hercules, CA, USA). All primary and secondary antibodies were obtained from Santa Cruz Biotechnology (Santa Cruz, CA, USA). Enhanced chemiluminescence kit was obtained from Bio-Rad (Hercules, CA, USA). All other chemicals used in the studies were of analytical or molecular biology grade unless otherwise specified.

### 2.2. Animals and Diets

Adult male Sprague-Dawley rats (150–200 g) obtained from the Animal Resource Centre (Canning Vale, WA, Australia) were used in the studies. Upon arrival, the rats were weight-matched and housed in polypropylene cages (3 per cage) to minimise isolation stress. The animal facility was well ventilated and maintained at an ambient temperature of 24 ± 2°C with 50–60% relative humidity with 12-hour light and dark cycle. The rats were acclimatised to the laboratory conditions for one week prior to experimentation and provided with standard diet and water ad libitum. Both the standard (catalogue no.: AIN93G) and the adenine (0.75% w/w) supplemented (catalogue no.: SF15-082) rat pellet diets were supplied by Speciality Feeds (Glen Forrest, WA, Australia). The standard diet contained (in weight percentage) approximately: 60% carbohydrate, 17.5% protein, 5% fat, 7% crude fibre, and the adenine-supplemented diet contained 0.75% adenine in the standard diet. The use and care of the animals in this experimental protocol was approved by the Institutional Animal Care and Ethics Committee (Approval Number: A11259) of the Western Sydney University, Australia, following the Australian National Health and Medical Research Council (NHMRC) guidelines on the “Australian Code of Practice for the Care and Use of Animals for Scientific Purposes.”

### 2.3. Experimental Design and Treatments

The rats were weight-matched and divided into three groups, each consisting of five to six rats. The control and CKD rats were fed with standard diet and adenine-supplemented diet (0.75% w/w adenine in standard diet), respectively, and treated with vehicle (1% sodium CMC) by oral gavage once daily for 24 days. The atorvastatin group (denoted as a CKD + ATV) was fed with adenine-supplemented diet and treated with atorvastatin at a dose of 10 mg/kg by oral gavage once daily for 24 days. On day 21, the rats were placed individually in metabolic cages, acclimatised for two days, and the 24-hour faeces and urine were collected on day 24. The urine samples were centrifuged at 1000 rpm for 10 minutes to remove food particles and debris and the supernatants were stored at −20°C until analysis. Thereafter, the rats were anesthetised with an intraperitoneal injection of ketamine (75 mg/kg) and xylazine (5 mg/kg) cocktail, and blood samples (approximately 3 mL) were collected from cardiac puncture and allowed to clot for 30 minutes before centrifuging at 3000 rpm for 15 minutes. The serum was separated and stored at −20°C until biochemical analysis. After blood collection, the liver, kidney, heart, skeletal muscle and epididymal fat tissue of each rat were immediately dissected, weighed and snap frozen in liquid nitrogen, and stored at −80°C until cellular and molecular studies. At the end of the procedure, the rats were euthanised by exsanguination from the abdominal aorta.

### 2.4. Biochemical Estimation

#### 2.4.1. Body Weight, Food Intake and Water Intake

The daily body weights were recorded in all groups of rats every day between 9 AM and 10 AM and continued up to 24 days. The 24-hour food and water intake from each cage were determined daily at 10 AM.

#### 2.4.2. Serum Biochemical Parameters

Serum total cholesterol, triglycerides, HDL cholesterol, NEFA, urea nitrogen, albumin and creatinine were estimated using the commercial diagnostic kits following the manufacturer's instructions. Serum very low density lipoprotein (VLDL) cholesterol and low density lipoprotein (LDL) cholesterol were calculated indirectly by Friedewald's equations [33]:

(1) $$\begin{array}{c} \text{VLDL}=\frac{\text{triglycerides}}{5},\\ \text{LDL}=\text{total cholesterol}-\left[\text{HDL}+\text{VLDL}\right]. \end{array}$$

Atherogenic index (AI) and coronary risk index [26] as measures of the extent of atherosclerotic lesions and coronary atherosclerosis development, respectively, were calculated using serum total cholesterol and HDL cholesterol of different groups of rats using the following mathematical formulae [34]:

(2) $$\begin{array}{c} \text{AI}=\frac{\left[\text{total cholesterol}-\text{HDL}\right]}{\text{HDL}},\\ \text{CRI}=\frac{\text{total cholesterol}}{\text{HDL}}. \end{array}$$

#### 2.4.3. Liver Lipid Content

Total lipids were extracted from the liver samples by the modified method of Hara and Radin [35] as described by Raubenheimer et al. [36]. Briefly, 75–100 mg aliquots of liver were homogenised in 20 volumes of isopropanol, shaken in an orbital shaker for 45 minutes and centrifuged at 3000 g for 15 minutes, and the supernatant was analysed for hepatic cholesterol, triglycerides and NEFA using commercial diagnostic kits following the manufacturer's instructions.

#### 2.4.4. Urine Biochemical Parameters

Total urinary protein (proteinuria) was estimated based on the method of Bradford following the manufacturer's instructions (Bio-Rad, Hercules, CA, USA), with absorbance measured at 595 nm using Thermo Multiskan microplate reader. Urine creatinine and urea nitrogen (UUN) were estimated using the commercial diagnostic kits following the manufacturer's instructions. Creatinine clearance was mathematically calculated as a clinical index of kidney function using serum and urine creatinine values as per the following formula [37]: creatinine clearance (mL/min/kg) = [urinary Cr (mg/dL) × urinary volume [38]/serum Cr (mg/dL)] × [1000/body weight (g)] × [1/1440 (min)].

#### 2.4.5. Faecal Lipid Content Estimation

The faeces samples collected during the last 24 hours of the treatment protocol were weighed and dried at 110°C for 24 hours. After drying and reweighing, the faeces samples were powdered using a mortar and stored at −20°C until analysis. The total lipids from rat faeces were extracted by the modified method of Folch et al. [39] as described by Kraus et al. [40]. Total cholesterol and triglycerides in rat faeces were estimated using an enzymatic colorimetric assay based on the cholesterol oxidase method following the manufacturer's instructions (PM Separations, USA), with absorbance measured at 550 nm using a UV-VIS spectrophotometer. The total bile acids from rat faeces were extracted by the method of De Wael et al. [41] and estimated using a colorimetric assay kit based on an enzymatic cholesterol method following the manufacturer's instructions (Wako Diagnostic, Japan), with absorbance measured at 560 nm using a UV-VIS spectrophotometer.

### 2.5. The Expression of Marker Proteins in the Various Tissues of CKD Rats

#### 2.5.1. Tissue Protein Extraction

Frozen tissue (i.e., liver, skeletal muscle, heart and adipose tissue) samples were homogenised on ice for 30 seconds using a *Tissue Master* homogeniser (Omni International, GA, USA) with five volumes of radio immunoprecipitation assay (RIPA) buffer (pH 8.0) containing 50 mM Tris, 150 mM NaCl, 1% Triton X-100, 0.5% sodium deoxycholate, 0.1% sodium dodecyl sulfate, and 10 *μ*L/mL protease and phosphatase inhibitors cocktail. The homogenates were centrifuged at 4°C at 10,000 g for 15 minutes, and the supernatants were collected. Protein concentrations were measured by the Bradford assay using bovine serum albumin as standard.

#### 2.5.2. Western Blot Analysis

The samples were mixed with loading buffer; proteins were denatured by heating at 95°C for 7 minutes, and 25 or 50 *μ*g of total protein was electrophoretically resolved on 8 to 10% Mini-PROTEAN® TGX™ Precast gels (Bio-Rad, Australia) at 120 V for 90 minutes and then transferred onto a nitrocellulose membrane (Bio-Rad, Australia) using a Trans-Blot Turbo® Transfer System (15 V for 30 min) or Mini Trans-Blot® Cell (100 V for 1 hour). After blotting, the membranes were blocked with 5% nonfat dry milk for 1 hour at room temperature. The membranes were then washed three times for 5 minutes each with Tris-buffered saline-0.1% Tween (TBST, pH 7.6) and incubated overnight at 4°C with mouse anti-HMG-CoA reductase (1 : 1000), mouse anti-LDLR (1 : 1000), goat anti-ACAT2 (1 : 1000), rabbit anti-hepatic lipase (1 : 1000), mouse anti-lipoprotein lipase (1 : 1000), mouse anti-VLDLR or mouse anti-LCAT (1 : 500), or goat anti-SR-B1 (1 : 500) antibody (Santa Cruz, Biotechnology, CA, USA) diluted with TBST. Blots were then again washed three times for 5 minutes each with TBST and incubated for 1 hour at room temperature with an appropriate horseradish peroxidase-conjugated secondary antibody (Santa Cruz Biotechnology, CA, USA) diluted at 1 : 10,000 with phosphate-buffered saline (PBS, pH 7.4). The membranes were again washed three times for 5 minutes each with TBST and incubated with enhanced chemiluminescence reagent (Clarity™ Western ECL, Bio-Rad, Australia) for 1 minute at room temperature. Immune complexes were detected after exposing the blots to ChemiDoc™ XRS system (Bio-Rad, Australia) at various time points. Quantitative image analysis was performed using NIH Image software (Image *J*) to determine the intensity of the protein signal, which was expressed relative to the amount of *β*-actin used as an internal control.

### 2.6. The Activity of Marker Enzymes in Various Tissues of CKD Rats

The activity of HMG-CoA reductase enzyme in the liver was assayed by the method of Rao and Ramakrishnan [42]. The enzyme activity of ACAT2 was performed using the fluorometric acetyltransferase activity assay kit (Abcam ab204536) as per the manufacturer's instructions, with fluorescence measured at Ex/Em = 380/520. The enzyme activity of LPL was performed using the fluorometric LPL activity assay kit (Roar Biomedical Inc) as per the manufacturer's instructions, with fluorescence measured at Ex/Em = 370/450. The LPL activity assay results were normalised to the amount of protein (nmol/mg of tissue or serum protein) and determined using the Bradford assay using bovine serum albumin as standard. The enzyme activity of LCAT was performed using the fluorometric LCAT activity assay kit (Roar Biomedical Inc.) as per the manufacturer's instructions, with fluorescence measured at two different wavelengths: (i) Ex/Em = 340/390 and (ii) Ex/Em = 340/470. The ratio of the two emission intensities (390 nm/470 nm) was used to express LCAT activity.

### 2.7. Data and Statistical Analysis

The results were expressed as means ± SEM To analyse the quantitative differences among the experimental groups, the data were subjected to the analysis of variance using the GraphPad 7.03 (GraphPad Software Inc., California, CA, USA) statistical software. Post hoc comparisons were made using Dunnett's multiple comparisons test. In all tests, *p* < 0.05 was used as the criterion for statistical significance.

## 3. Results

### 3.1. General Laboratory Data

There was no significant difference in the initial body weights (194.2 ± 5.77 g to 198.83 ± 2.41 g, day 0 body weight, data not shown in Table 1) between different experimental groups. However, by the end of week 1, CKD rats exhibited significant decrease in body weight compared to control rats (data not shown) and this difference continued till the end of the 24-day treatment period (Table 1). However, the group treated with atorvastatin (10 mg/kg) did not show a significant improvement in body weight compared with CKD rats at the end of the 24-day treatment period. CKD rats significantly increased water intake compared with control rats at the end of the 24-day treatment period, while atorvastatin-treated rats showed significant reduction in water intake compared with CKD rats. In addition, the mean daily food intake was significantly decreased in CKD and atorvastatin-treated rats compared to control rats. Nevertheless, the mean daily food intake was not significantly different between CKD and atorvastatin-treated rats. Moreover, the higher urinary volume of CKD rats, which is hallmark of the renal damage, was significantly attenuated by atorvastatin treatment. In addition, right and left kidney weights of CKD rats (normalised to body weight) were significantly increased compared to those of the control rats. However, CKD-induced kidney weight gain was significantly reduced by atorvastatin treatment.

### 3.2. Serum, Tissue and Faecal Lipid Data

The serum, hepatic, renal and faecal lipid profile data are shown in Table 2. The CKD rats exhibited a significant increase in serum total cholesterol, LDL cholesterol, VLDL cholesterol, triglycerides, AI, CRI and NEFA and decrease in serum HDL cholesterol. Chronic atorvastatin administration resulted in a significant reduction in serum total cholesterol, LDL cholesterol, VLDL cholesterol, triglycerides, AI, CRI and NEFA but did not significantly increase serum HDL cholesterol compared to CKD rats. The CKD rats had significant elevation of cholesterol, triglycerides, and NEFA in the liver which was significantly reduced by atorvastatin treatment. Moreover, faecal total lipids, cholesterol, triglycerides, and total bile acids were not changed among the experimental groups.

### 3.3. Kidney Function Tests

As expected, the CKD rats had a significant elevation of serum creatinine and blood urea nitrogen concentrations and urinary protein excretion (14-fold) compared to control rats. Furthermore, CKD rats exhibited a significant decrease of urine creatinine, urine urea nitrogen, creatinine clearance and serum albumin levels. Atorvastatin treatment significantly lowered serum creatinine, blood urea nitrogen concentrations and urinary protein excretion and significantly elevated urine creatinine, urine urea nitrogen, creatinine clearance and serum albumin levels compared to CKD rats (Table 3).

### 3.4. Cellular Effects of Atorvastatin on HMG-CoA Reductase, ACAT2 and LDL Receptor in the Liver of CKD Rats

**Figure 1:**
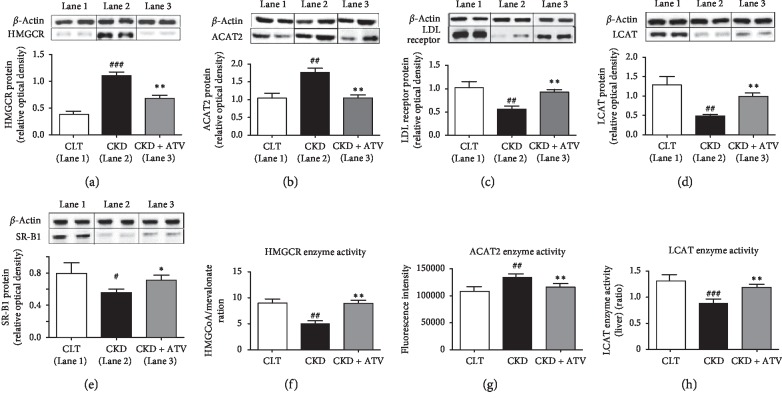
Effects of atorvastatin on expression and enzymatic activity of various proteins in the liver of adenine-induced CKD rats. (a) HMG-CoA reductase (HMGCR), (b) ACAT2, (c) LDL receptor (LDLr), (d) LCAT, and (e) SR-B1 protein expression as determined by western blot analysis in liver of rats treated for 24 days either with normal diet (control) or adenine-supplemented diet (CKD) alone or with atorvastatin (CKD + ATV). Enzyme activity of (f) HMG-CoA reductase, (g) ACAT2, and (h) LCAT in the liver of control, CKD, and atorvastatin-treated rats. Relative optical density was calculated relative to *β*-actin used as internal control. Each bar represents the mean ± SEM of 5–6 rats calculated. Significant difference from control: ^#^*p* < 0.05, ^##^*p* < 0.01, and ^###^*p* < 0.001. Significant difference from CKD: ^*∗*^*p* < 0.05 and ^*∗∗*^*p* < 0.01.

We have shown that chronic atorvastatin administration reduced serum and hepatic cholesterol and LDL cholesterol in CKD rats. As expected, HMG-CoA reductase and ACAT2 abundance and activity were significantly increased in the liver of CKD rats than those found in the control rats. This was associated with a significant reduction of LDL receptor abundance in the liver tissues of CKD rats compared to the corresponding values found in the control rats. The administration of atorvastatin normalised the HMG-CoA and ACAT2 expression and enzyme activity in the liver of CKD rats. Atorvastatin also significantly increased the LDL receptor protein expression in the liver when compared to CKD rats (Figure 1).

### 3.5. Cellular Effects of Atorvastatin on LCAT and SR-B1 in the Liver of CKD Rats

In the present study, atorvastatin showed a tendency to improve the HDL cholesterol in the serum of CKD rats but did not reach significance. However, atherogenic and coronary risk indices (HDL to total cholesterol ratio) were significantly decreased upon atorvastatin treatment. Therefore, we investigated the underlying molecular mechanism responsible for the observed effects by measuring the expression and enzyme activity of the marker protein responsible for HDL synthesis in the liver of CKD rats (Figure 1). Our investigation revealed that LCAT and SR-B1 (HDL receptor) were significantly decreased in the liver of untreated CKD rats. Atorvastatin administration improved the LCAT and SR-B1 protein abundance. Furthermore, atorvastatin also improved the suppressed LCAT enzyme activity in the liver of CKD rats (Figure 1).

### 3.6. Cellular Effects of Atorvastatin on Lipase and VLDL Receptor in Various Tissues of CKD Rats

**Figure 2:**
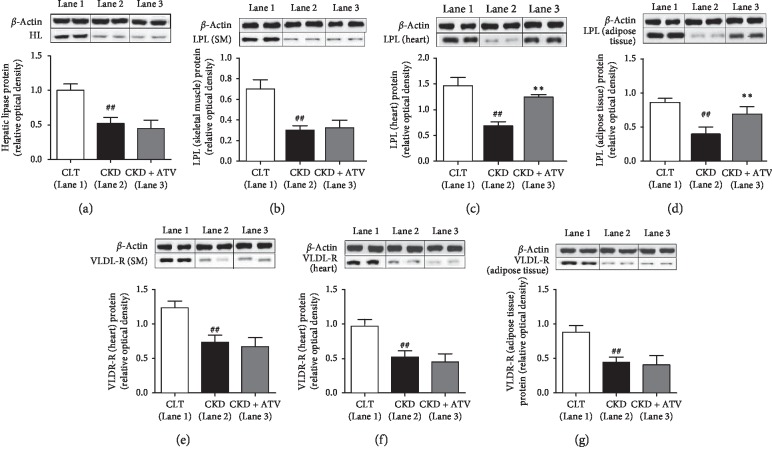
Effects of atorvastatin on protein expression of lipase and VLDL receptor in various tissues of adenine-induced CKD rats. Lipase expression in (a) liver, (b) skeletal muscle, (c) heart, and (d) adipose tissue and VLDL receptor expression in (e) skeletal muscle, (f) heart, and (g) adipose tissue as determined by western blot analysis in liver of rats treated for 24 days either with normal diet (control) or adenine-supplemented diet (CKD) alone or with atorvastatin (CKD + ATV). Relative optical density was calculated relative to *β*-actin used as internal control. Each bar represents the mean ± SEM of 5–6 rats. Significant difference from control: ^##^*p* < 0.01. Significant difference from CKD: ^*∗∗*^*p* < 0.01. SM, skeletal muscle; AT, adipose tissue; HL, hepatic lipase; LPL, lipoprotein lipase.

**Figure 3:**
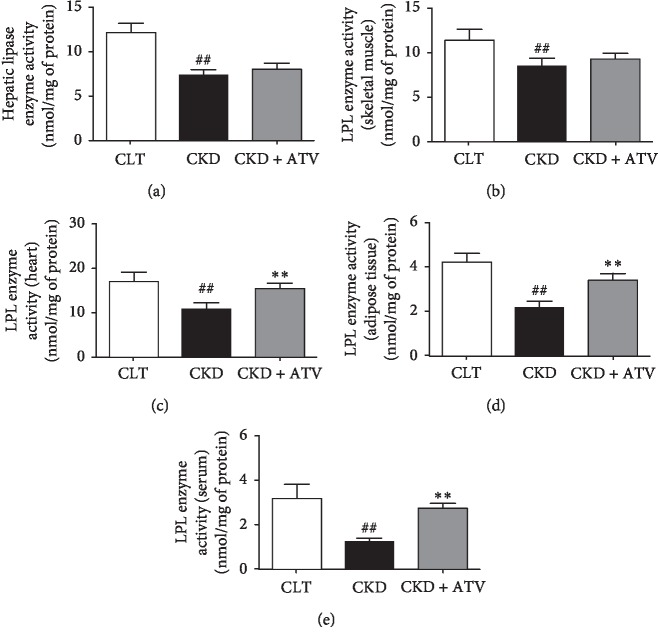
Effects of atorvastatin on enzymatic activity of lipase in various tissues and serum of adenine-induced CKD rats. Lipase enzymatic activity in (a) liver, (b) skeletal muscle, (c) heart, (d) adipose tissue, and (e) serum of rats treated for 24 days either with normal diet (control) or adenine-supplemented diet (CKD) alone or with atorvastatin (CKD + ATV). Each bar represents the mean ± SEM of 5–6 rats. Significant difference from control: ^##^*p* < 0.01. Significant difference from CKD: ^*∗∗*^*p* < 0.01.

Our results indicate that long-term atorvastatin administration reduced serum and hepatic triglycerides and triglyceride-rich lipoprotein (VLDL) and thereby, effectively reduced CKD-induced hypertriglyceridemia in CKD rats. It can be postulated that the observed triglycerides lowering effects could be due to lipase and VLDL receptor deficiencies in various tissues such as liver, skeletal muscle, heart and adipose tissue. The underlying molecular mechanisms of atorvastatin were studied by measuring the lipase and VLDL receptor protein expression in the liver, skeletal muscle, heart and adipose tissue of CKD rats. Furthermore, we have measured the enzyme activity of lipase in the liver, heart, skeletal muscle, adipose tissue and serum of CKD rats. The western blot data are shown in Figure 2. In agreement with previous studies [13–15], the lipase abundance was significantly reduced in the liver (hepatic lipase), skeletal muscle, heart and adipose tissue (lipoprotein lipase) of the untreated CKD rats when compared with the control rats. This was accompanied by the marked reduction of VLDL receptor in the skeletal muscle, heart and adipose tissue of the untreated CKD rats compared to control rats. Atorvastatin supplementation normalised the lipase expression in the liver (hepatic lipase), skeletal muscle, heart and adipose tissue. Moreover, atorvastatin did not alter the VLDL receptor protein expression in the skeletal muscle, heart and adipose tissue compared to untreated CKD rats. Furthermore, lipase enzyme activity in various tissues as well as in the serum was significantly reduced in CKD rats. However, atorvastatin increased the lipase enzyme activity in the liver, skeletal muscle, heart, adipose tissue and serum (Figure 3).

## 4. Discussion

In the present study, the protective effects of atorvastatin were examined on hepatic lipid derangement and renal damage in an adenine-induced rat model of CKD. Long-term feeding of adenine is known to suppress the excretion of various nitrogenous compounds due to renal tubular occlusion and produce metabolic abnormalities in rats that closely mimic CKD in humans [43]. To our knowledge, this is the first study to use an adenine-induced rat model of CKD to investigate the protective effects of atorvastatin on hepatic lipid metabolism.

The untreated CKD rats exhibited a 2-fold rise in serum and hepatic total cholesterol and a nearly 4-fold increase in serum LDL cholesterol concentration. This was associated with a marked reduction in hepatic tissue LDL receptor abundance confirming earlier reports in the rats with 5/6 nephrectomy-induced CKD [44] and in the rats with puromycin-induced nephrotic syndrome [45]. Atorvastatin administration resulted in a marked reduction in serum total and LDL cholesterol concentration. This was accompanied by the amelioration of hepatic LDL receptor deficiency. The decline in serum LDL cholesterol in atorvastatin-treated CKD rats could be, at least in part, due to attenuation of LDL receptor deficiency and restoration of hepatic LDL receptor. The mechanism by which atorvastatin reverses hepatic LDL cholesterol in CKD is unclear. However, it may represent a compensatory response to the inhibition of cellular cholesterol biosynthesis via HMG-CoA reductase inhibition. The untreated CKD rats in the present study exhibited a significant decrease in serum HDL cholesterol and significantly increase serum total cholesterol-to-HDL cholesterol, representing atherogenic and coronary risk profile. This was also associated with the severe reduction in hepatic LCAT activity and LCAT and HDL receptor (SR-B1) protein expression. These results confirm the earlier reports in animal and humans with CKD [16, 18, 46]. LCAT and HDL receptor deficiencies can be responsible for impaired HDL maturation, defective reverse cholesterol transportation, and atherogenic and coronary risk profile in patients and CKD animals. Atorvastatin administration for 24 days reversed, at least in part, CKD-induced LCAT deficiency as evident by increased hepatic LCAT activity and protein expression. Moreover, the hepatic HDL receptor was also normalised by atorvastatin administration. The correction of LCAT and HDL receptor deficiencies was most likely responsible for the trend (but not significant) in the rise of serum HDL cholesterol levels observed in atorvastatin-treated CKD animals. In addition, the amelioration of proteinuria (which is the defining feature of CKD) with atorvastatin administration must have contributed to the observed improvements of the associated LDL and HDL receptor deficiencies. Therefore, the alleviation of CKD-induced altered cholesterol metabolism in the present study by atorvastatin could be, at least in part, due to its renoprotective effects, which needs further investigation.

ACAT catalyses intracellular esterification of cholesterol, and as such involved in the intestinal absorption of cholesterol, hepatic assembly of apoB-containing lipoproteins, foam cell formation and atherogenesis [47]. In agreement with previous studies [17, 48], the CKD rats employed in this study showed marked upregulation of liver-specific ACAT2 activity and expression, which significantly decreased upon atorvastatin administration. Increased ACAT2 enzyme activity and protein expression enhanced the esterification of free cholesterol in the endoplasmic reticulum. This further reduced the total free cholesterol pool which, in turn, resulted in the upregulation of HMG-CoA reductase and, hence, increased cholesterol biosynthesis (also exhibited in our untreated CKD rats). However, HMG-CoA reductase inhibition by atorvastatin could be the reason of the observed ACAT2 activity and protein expression inhibition, but this needs further investigation.

In accordance with previous studies, our untreated CKD rats displayed the marked elevation of serum triglycerides, NEFA, and VLDL levels. Furthermore, hepatic triglycerides and NEFA levels were also significantly higher in CKD rats. This was associated with a significant reduction in lipase enzyme activity and protein expression in the liver (hepatic lipase), heart, skeletal muscle, adipose tissue and serum. Furthermore, the CKD rats in this study showed marked downregulation of VLDL receptor in these tissues. Thus, the results presented in this study substantiate the claim that CKD-induced hypertriglyceridemia could be due, at least in part, to lipase deficiency [49, 50]. Long-term atorvastatin administration in the CKD rats results in a significant decline in serum and hepatic triglyceride and NEFA levels, as well as serum VLDL levels. This was accompanied by the upregulation of lipase protein expression and enzyme activity in hepatic and various extrahepatic tissues. Furthermore, hypertriglyceridemia in CKD rats is associated with impaired maturation of HDL-3 to HDL-2 mediated by LCAT deficiency which further contributes to defective lipolysis and clearance of triglyceride-rich lipoproteins (i.e. VLDL and chylomicrons) in circulation. Thus, it can be postulated that improvement in hypertriglyceridemia in the atorvastatin-treated CKD rats could be due to the improvement in HDL metabolism. Several clinical trials of statins have shown the beneficial effects on serum LDL, HDL and triglyceride in nonuremic hyperlipidemic population [51]. Thus, the effects observed in the CKD rats are consistent with those founds in hyperlipidemic population.

## 5. Conclusion

In conclusion, CKD induced by an adenine-modified diet in normal rats leads to the derangement of lipid metabolism. This is associated with the dysregulation of cholesterol and triglyceride-rich lipoprotein metabolism. Long-term statin administration to CKD animals corrects the observed dyslipidaemia by improving cholesterol and triglyceride metabolism. Furthermore, the present study provides evidence of the renoprotective effects of atorvastatin in CKD animals. We conclude that the present findings provide the rational basis that the renoprotective strategies could be effective in retarding the altered lipid metabolism in CKD patients.

## Figures and Tables

**Table 1:** Effect of atorvastatin treatment on general laboratory parameters after 24 days in adenine-induced CKD rats.

	CLT	CKD	CKD + ATV
Body weight (g)	380.4 ± 6.04	218.08 ± 5.22^###^	231 ± 7.61
Average water intake (mL/day)	41.58 ± 0.59	52.91 ± 1.13^###^	46.22 ± 0.35^*∗∗∗*^
Average food intake (g/day)	29.04 ± 2.24	18.78 ± 0.41^###^	18.38 ± 0.18
Urinary volume (mL/24 h)	24.40 ± 2.21	37.00 ± 1.58^###^	27.83 ± 2.02^*∗∗*^
L. kidney weight (g/100 g body wt)	0.43 ± 0.02	2.44 ± 0.08^###^	1.60 ± 0.11^*∗∗∗*^
R. kidney weight (g/100 g body wt)	0.41 ± 0.02	2.38 ± 0.11^###^	1.46 ± 0.12^*∗∗∗*^

Values represent the mean ± SEM of 5 to 6 rats (*n* = 5 to 6). Significant difference from control: ^###^*p* < 0.001. Significant difference from CKD: ^*∗∗*^*p* < 0.01, ^*∗∗∗*^*p* < 0.001. ATV: atorvastatin; CLT: control; CKD: chronic kidney disease.

**Table 2:** Effect of atorvastatin treatment on lipid parameters of various biological samples after 24 days in adenine-induced CKD rats.

	CLT	CKD	CKD + ATV
Serum total cholesterol (mg/dL)	82.11 ± 8.38	153.28 ± 4.64^###^	97.30 ± 11.32^*∗∗*^
Serum LDL cholesterol (mg/dL)	32.97 ± 14.41	115.28 ± 5.23^###^	60.56 ± 13.08^*∗∗∗*^
Serum VLDL cholesterol (mg/dL)	13.72 ± 0.76	21.84 ± 0.34^###^	14.02 ± 1.94^*∗∗∗*^
Serum HDL cholesterol (mg/dL)	35.42 ± 3.10	16.16 ± 3.33^###^	22.08 ± 2.80^ns^
Serum triglycerides (mg/dL)	68.59 ± 3.82	109.19 ± 1.71^###^	70.09 ± 9.71^*∗∗∗*^
Serum NEFA (*μ*Eq/L)	872.63 ± 18.03	1099.16 ± 16.36^#^	767.41 ± 92.22^*∗∗*^
Atherogenic index	1.31 ± 0.17	10.63 ± 2.28^###^	3.88 ± 0.93^*∗∗∗*^
Coronary risk index	2.31 ± 0.17	11.63 ± 2.28^###^	4.88 ± 0.93^*∗∗∗∗*^
Liver cholesterol (mg/g of liver)	0.38 ± 0.07	4.95 ± 1.30^###^	2.42 ± 0.44^*∗∗∗*^
Liver triglycerides (mg/g of liver)	2.55 ± 0.73	10.51 ± 1.39^###^	4.09 ± 1.32^*∗∗∗*^
Liver NEFA (mg/g of kidney)	0.73 ± 0.05	1.20 ± 0.06^##^	0.97 ± 0.15^ns^
Total faecal lipid (mg/g of dried faeces)	44.82 ± 3.9	51.33 ± 7.80	46.33 ± 0.63^ns^
Faecal cholesterol (mg/g of dried faeces)	1.33 ± 0.16	2.21 ± 0.66	2.26 ± 0.22^ns^
Faecal triglyceride (mg/g of dried faeces)	3.21 ± 0.33	3.71 ± 0.53	3.24 ± 0.15^ns^
Total bile acid (*μ*mol/g of dried faeces)	46.23 ± 1.42	52.13 ± 6.25	54.30 ± 5.86^ns^

Values represent the mean ± SEM of 5 to 6 rats (*n* = 5 to 6). Significant difference from control: ^#^*p* < 0.05 and ^###^*p* < 0.001. Significant difference from CKD: ^*∗∗*^*p* < 0.01 and ^*∗∗∗*^*p* < 0.001. No significant difference from CKD: ns; CLT: control; CKD: chronic kidney disease.

**Table 3:** Effect of atorvastatin treatment on kidney biochemical parameters after 24 days in the serum and urine of adenine-induced CKD rats.

	CLT	CKD	CKD + ATV
Serum creatinine (mg/dL)	0.45 ± 0.07	2.62 ± 0.08^###^	1.49 ± 0.26^*∗*^
Urine creatinine (mg/dL)	28.69 ± 2.61	11.23 ± 0.34^###^	19.86 ± 0.91^*∗∗∗*^
Creatinine clearance (ml/min/kg)	3.07 ± 0.20	0.63 ± 0.13^###^	1.29 ± 0.25^*∗∗*^
Blood urea nitrogen (mg/dL)	4.46 ± 1.53	13.09 ± 1.19^##^	5.95 ± 0.75^*∗∗*^
Urine urea nitrogen (mg/dL)	14.28 ± 1.06	5.95 ± 0.75^###^	10.11 ± 1.10^*∗∗*^
Serum albumin (mg/dL)	8.41 ± 0.32	5.47 ± 0.08^###^	6.70 ± 0.29^*∗*^
Urine protein (mg/24 h)	5.52 ± 0.83	70.73 ± 4.14^###^	9.71 ± 2.60^*∗∗∗*^

Values represent the mean ± SEM of 5 to 6 rats (*n* = 5 to 6). Significant difference from control: ^##^*p* < 0.01 and ^###^*p* < 0.001. Significant difference from CKD:^*∗∗*^*p* < 0.05, ^*∗∗*^*p* < 0.01, and ^*∗∗∗*^*p* < 0.001. CLT: control; CKD: chronic kidney disease.

## Data Availability

The data used to support the findings of this study are included within the article.
